# Supplementary material for: Small-scale protocols to characterize mitochondrial Complex V activity and assembly in peripheral blood mononuclear cells
Source: PLoS One. 2025 May 8;20(5):e0323136. doi: 10.1371/journal.pone.0323136 (PMC12061129; doi:10.1371/journal.pone.0323136)
Supplement: S1 Table — (PDF) [file pone.0323136.s001.pdf]

S1 Table. Age and sex of PBMC donors.

PBMC donor	Age (y)	Sex
C1	48	Female
C2	48	Male
C3	24	Female
C4	28	Male
C5	36	Female
C6	66	Male
