# Supplementary material for: Small-scale protocols to characterize mitochondrial Complex V activity and assembly in peripheral blood mononuclear cells
Source: PLoS One. 2025 May 8;20(5):e0323136. doi: 10.1371/journal.pone.0323136 (PMC12061129; doi:10.1371/journal.pone.0323136)
Supplement: S1 Fig — Measurements of 4 × 10^5^ PBMCs solubilized with 0.01% of digitonin. Two separate experiments are shown. (PDF) [file pone.0323136.s002.pdf]

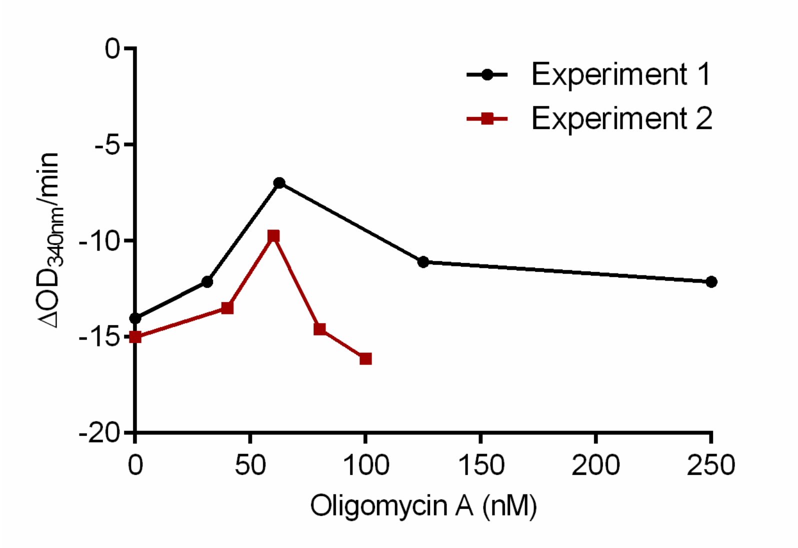

S1 Fig. Effect of increasing oligomycin A concentrations on the decrease in OD_{320nm} (ΔOD_{320nm}) per minute in spectrophotometric ATPase activity assays of PBMCs. Measurements of 4 × 10^5 PBMCs solubilized with 0.01% of digitonin. Two separate experiments are shown.
