# Supplementary material for: Small-scale protocols to characterize mitochondrial Complex V activity and assembly in peripheral blood mononuclear cells
Source: PLoS One. 2025 May 8;20(5):e0323136. doi: 10.1371/journal.pone.0323136 (PMC12061129; doi:10.1371/journal.pone.0323136)
Supplement: S2 Fig — Measurements of 4 × 10^5^ PBMCs without oligomycin A. Two separate experiments are shown. (PDF) [file pone.0323136.s003.pdf]

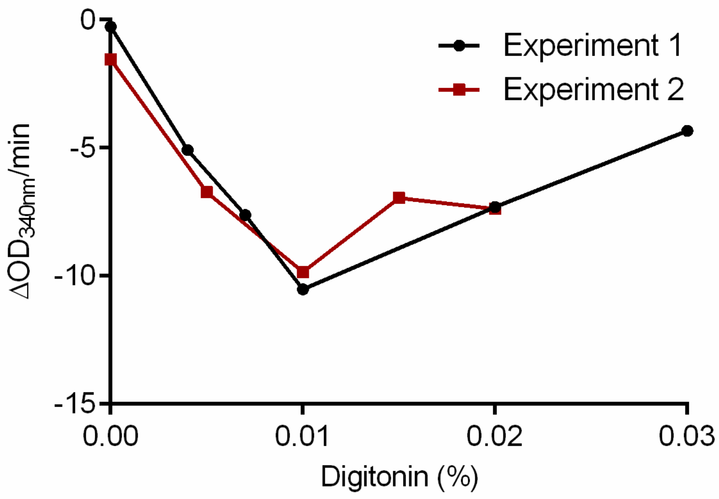

S2 Fig. Effect of increasing digitonin concentrations on the decrease in OD_{320nm} (ΔOD_{320nm}) per minute in spectrophotometric ATPase activity assays of PBMCs. Measurements of 4×10^5 PBMCs without oligomycin A. Two separate experiments are shown.
