# Supplementary material for: Small-scale protocols to characterize mitochondrial Complex V activity and assembly in peripheral blood mononuclear cells
Source: PLoS One. 2025 May 8;20(5):e0323136. doi: 10.1371/journal.pone.0323136 (PMC12061129; doi:10.1371/journal.pone.0323136)
Supplement: S3 Fig — PBMCs were solubilized with 0.01% of digitonin. (PDF) [file pone.0323136.s004.pdf]

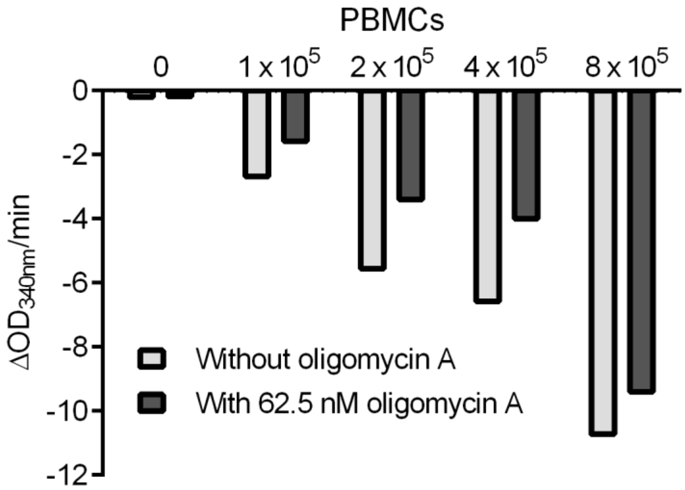

S3 Fig. Effect of increasing PBMC cell numbers on the decrease in OD_{320nm} (ΔOD_{320nm}) per minute in spectrophotometric ATPase activity assays measured without or with 62.5 nM oligomycin A. PBMCs were solubilized with 0.01% of digitonin.
