# Supplementary material for: Small-scale protocols to characterize mitochondrial Complex V activity and assembly in peripheral blood mononuclear cells
Source: PLoS One. 2025 May 8;20(5):e0323136. doi: 10.1371/journal.pone.0323136 (PMC12061129; doi:10.1371/journal.pone.0323136)
Supplement: S4 Fig — The F1 and Fo moieties of CX-V are outlined. Pi, inorganic orthophosphate. (PDF) [file pone.0323136.s005.pdf]

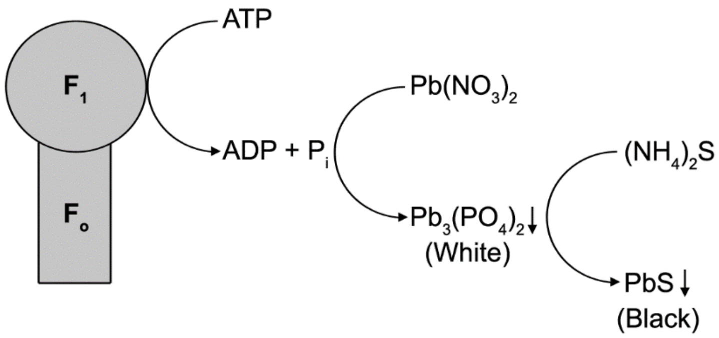

S4 Fig. Chemical reactions of the in-gel CX-V staining procedure. The F_1 and F_o moieties of CX-V are outlined. P_i, inorganic orthophosphate.
