# Supplementary material for: Small-scale protocols to characterize mitochondrial Complex V activity and assembly in peripheral blood mononuclear cells
Source: PLoS One. 2025 May 8;20(5):e0323136. doi: 10.1371/journal.pone.0323136 (PMC12061129; doi:10.1371/journal.pone.0323136)
Supplement: S5 Fig — (A) In-gel CX-V activity staining of extracts from increasing numbers of PBMCs. (B) Quantification of the in-gel CX-V activity signals in extracts from increasing numbers of PBMCs. Note the distortion of the band when overloaded with an extract derived from 9 × 10^5^ PBMCs. (PDF) [file pone.0323136.s006.pdf]

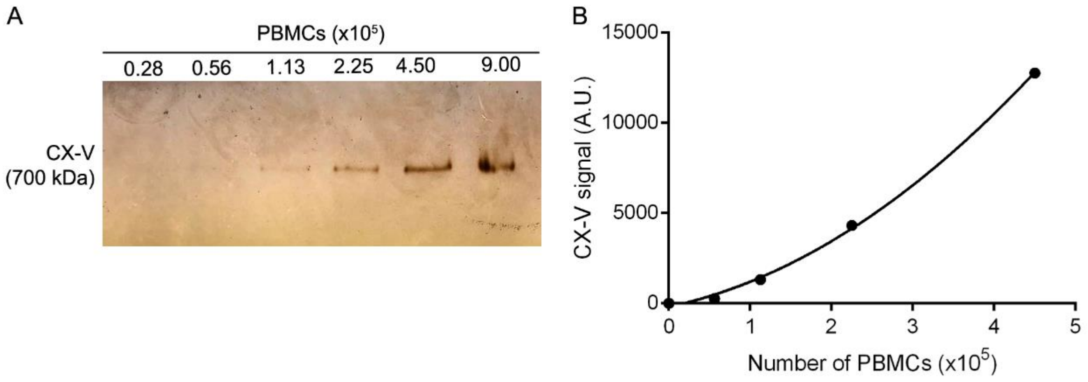

S5 Fig. Determination of the detection limit of in-gel CX-V activity staining. (A) In-gel CX-V activity staining of extracts from increasing numbers of PBMCs. (B) Quantification of the in-gel CX-V activity signals in extracts from increasing numbers of PBMCs. Note the distortion of the band when overloaded with an extract derived from 9 × 10^5 PBMCs.
