# Supplementary material for: Small-scale protocols to characterize mitochondrial Complex V activity and assembly in peripheral blood mononuclear cells
Source: PLoS One. 2025 May 8;20(5):e0323136. doi: 10.1371/journal.pone.0323136 (PMC12061129; doi:10.1371/journal.pone.0323136)
Supplement: S6 Fig — (PDF) [file pone.0323136.s007.pdf]

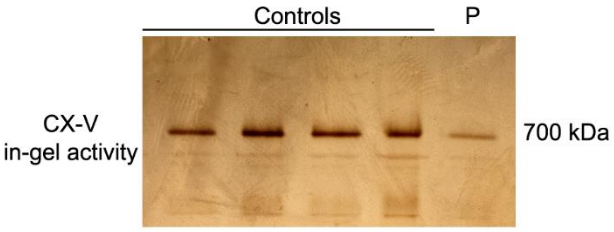

S6 Fig. In-gel CX-V activity of 15 μg protein extracts from cultured fibroblast of four control subjects and one patient (P) with a mitochondrial translation deficiency.
