# Supplementary figures and images for: Small-scale protocols to characterize mitochondrial Complex V activity and assembly in peripheral blood mononuclear cells
Source: PLoS One. 2025 May 8;20(5):e0323136. doi: 10.1371/journal.pone.0323136 (PMC12061129; doi:10.1371/journal.pone.0323136)

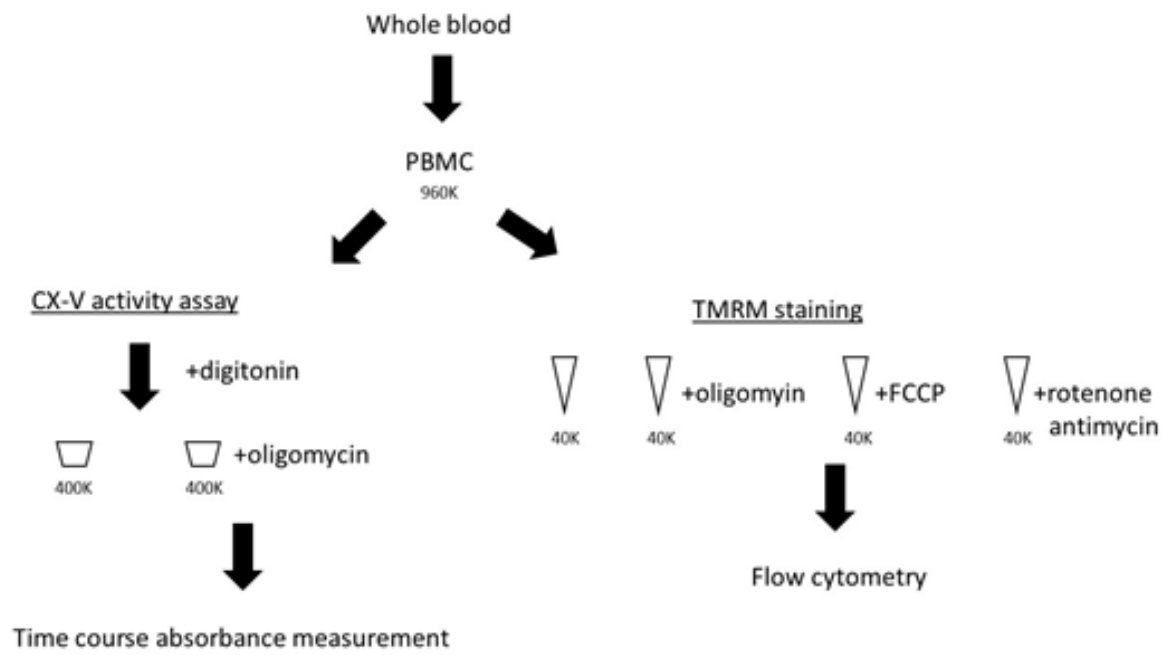

S7 Fig. Workflow of the spectrophotometric CX-V assay and  $\Delta\Psi_m$  analysis

Supplement: S7 Fig — (PDF) [file pone.0323136.s008.pdf]

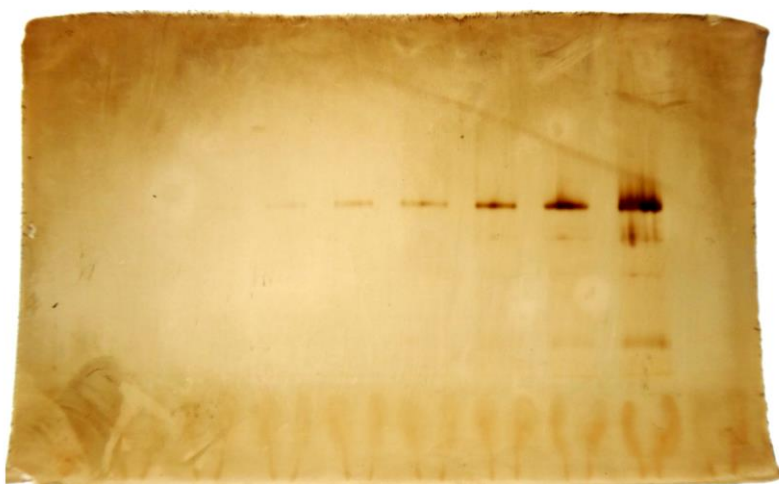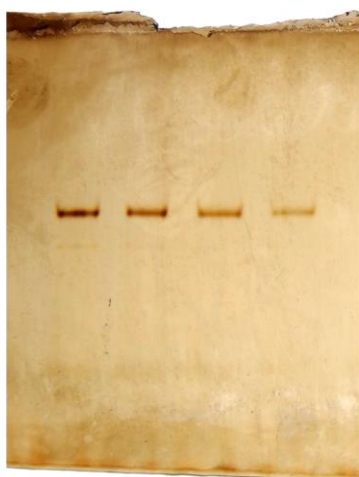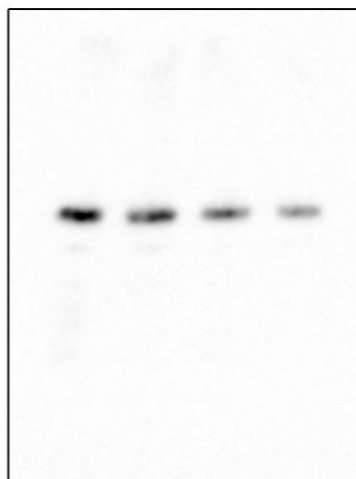

**S2 Raw images. Uncropped, unadjusted images of in-gel activity staining and western blot.**

Supplement: S2 Raw images — (PDF) [file pone.0323136.s011.pdf]
