# Supplementary material for: Small-scale protocols to characterize mitochondrial Complex V activity and assembly in peripheral blood mononuclear cells
Source: PLoS One. 2025 May 8;20(5):e0323136. doi: 10.1371/journal.pone.0323136 (PMC12061129; doi:10.1371/journal.pone.0323136)
Supplement: S1 Raw images — Left panel: raw scans upon different treatments. A gate was applied to cells >6 μm (X-axis, linear scale) and 2 log fluorescence (Y-axis, log scale). Right panel: Comparisons were made between untreated PBMCs (UT, grey background) and PBMCs with added oligomycin A (blue curves), rotenone+antimycin A (red curves) or FCCP (pink curves) drawn in a histogram (fluorescence versus cell number). (PDF) [file pone.0323136.s010.pdf]

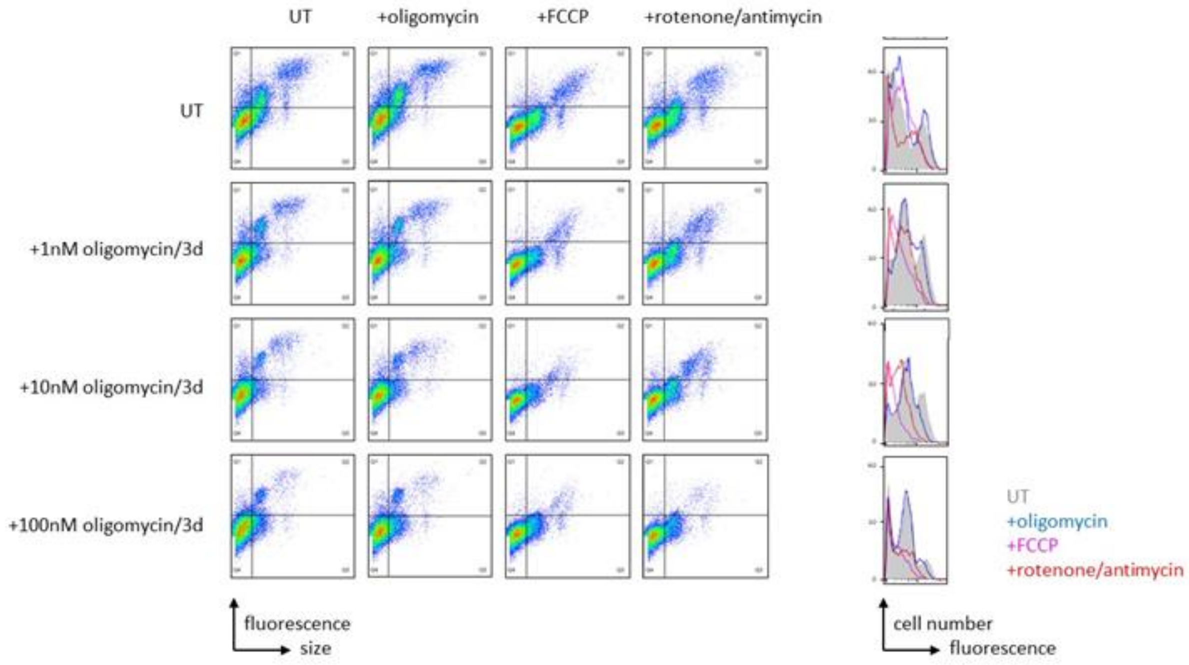

S1 Raw images. Flow cytometry graphs upon TMRM staining. Left panel: raw scans upon different treatments. A gate was applied to cells >6 μm (X-axis, linear scale) and 2 log fluorescence (Y-axis, log scale). Right panel: Comparisons were made between untreated PBMCs (UT, grey background) and PBMCs with added oligomycin A (blue curves), rotenone+antimycin A (red curves) or FCCP (pink curves) drawn in a histogram (fluorescence versus cell number).
